# Cytoreductive Radical Prostatectomy in Oligometastatic Prostate Cancer and the Role of the Robotic Platform: A Narrative Review

**DOI:** 10.3390/cancers18193207

**Published:** 2026-10-04

**Authors:** Juan Gomez Rivas, Pedro Gabriel Silva, Helena Margot Flôres Soares da Silva, Luís Severo, Luís Campos Pinheiro, Isabel Galante Romo, Enrique Redondo-González, Claudia González-Santander, Jesús Moreno Sierra

**Affiliations:** 1 Department of Urology, Hospital Clínico San Carlos, Instituto de Investigación Sanitaria del Hospital Clínico San Carlos (IdISSC), Universidad Complutense de Madrid, 28040 Madrid, Spain; m.isabel.galante@gmail.com (I.G.R.); eredon01@ucm.es (E.R.-G.); claudiaglezsantander@gmail.com (C.G.-S.); jemoreno@ucm.es (J.M.S.); 2 Department of Urology, Unidade Local de Saúde de São José, 1150-199 Lisbon, Portugal; luis.severo@ulssjose.min-saude.pt (L.S.); campos.pinheiro@ulssjose.min-saude.pt (L.C.P.); 3 Faculdade de Medicina, Universidade Federal do Rio Grande do Sul, Porto Alegre 90035-003, Brazil; helena.margot0403@gmail.com

**Keywords:** oligometastatic prostate cancer, robot-assisted radical prostatectomy, cytoreductive prostatectomy, local therapy, PSMA PET, multimodal treatment, metastatic hormone-sensitive prostate cancer

## Abstract

**Background**: Oligometastatic prostate cancer (OMPC) comprises biologically and clinically distinct states with a limited number of detectable metastases. Improved systemic therapy and prostate-specific membrane antigen positron emission tomography/computed tomography (PSMA PET/CT) have intensified interest in treating the primary tumor. **Methods**: In this narrative review we searched PubMed/MEDLINE from inception to 1 July 2026, reviewed current guidelines, and searched ClinicalTrials.gov, the ISRCTN registry and the EU Clinical Trials Register for ongoing surgical studies, prioritizing randomized trials, prospective studies, PSMA PET-staged cohorts, systematic reviews, guidelines, and studies of perioperative, functional and patient-reported outcomes. **Results**: Registry and institutional data generated the hypothesis that cytoreductive prostatectomy improves outcomes but are vulnerable to selection bias; prospective and PSMA PET-staged cohorts established feasibility. The phase II trial by Dai and colleagues supported radical treatment of the primary tumor but combined surgery and radiotherapy. RAMPP provided the first prostatectomy-specific randomized signal, with lower 5-year cancer-specific mortality after radical prostatectomy plus best systemic therapy than after systemic therapy alone (13% versus 23%; hazard ratio 0.39, 95% confidence interval 0.16–0.98), but accrual ended early, clinical progression was similar, overall survival was not significantly improved, systemic therapy predated contemporary intensification, and major surgery-related complications occurred in 14%. A randomized phase II trial with a predominantly surgical local-therapy arm found no progression-free or overall survival benefit, so randomized evidence is mixed. All randomized evidence concerns radical prostatectomy or local treatment irrespective of surgical approach; no trial has tested a robot-specific oncologic effect. Guidelines recommend prostate radiotherapy for selected low-volume de novo metastatic disease and restrict surgery to clinical trials. **Conclusions**: Cytoreductive prostatectomy, delivered mostly as robot-assisted radical prostatectomy (RARP), is feasible and may improve local control while providing complete pathology, but it remains investigational; the robotic approach is the surgical platform, not a modality with distinct oncologic efficacy. Appropriate use requires expert-center surgery, modern staging, guideline-concordant systemic therapy, multidisciplinary selection, transparent counseling and, preferably, trial participation. Future trials must establish whether prostatectomy adds meaningful benefit beyond modern systemic therapy, prostate radiotherapy and metastasis-directed treatment, while measuring morbidity, continence and quality of life.

## Simple Summary

Oligometastatic prostate cancer is usually treated with hormone-based drugs and, in selected men, with radiotherapy. Whether radical prostatectomy, today most often with robot-assisted techniques, adds any benefit is uncertain. This narrative review summarizes the available evidence, from registry data to recent randomized trials, on radical prostatectomy in men with limited metastatic disease. Surgery is feasible in expert hands, may reduce local complications and provides complete pathological information. One randomized trial suggested fewer cancer deaths after surgery, but another found no benefit, overall survival was not clearly improved, and surgery carries risks of complications. Current guidelines therefore restrict surgery in metastatic disease to clinical trials. We propose an explicitly investigational pathway for patient selection and outline the ongoing trials that could help settle the question.

## 1. Introduction

Oligometastatic prostate cancer (OMPC) is not a single disease entity but an operational description for men with a limited number of detectable metastases, commonly one to five, whose prognosis may differ from that of extensive metastatic disease. It spans de novo synchronous metastases, metachronous recurrence, nodal-only and bone-predominant disease, oligoprogression, and disease defined by conventional or molecular imaging—distinctions that influence prognosis, treatment intent, and the plausibility of benefit from treating the primary tumor [1,2,3,4].

Modern systemic therapy remains the foundation of treatment for metastatic hormone-sensitive prostate cancer (mHSPC), and guidelines recommend combination therapy rather than androgen deprivation therapy (ADT) monotherapy for fit patients [5,6,7]. Local treatment must therefore be judged as an addition to effective systemic therapy, not a substitute—a principle especially important when interpreting older surgical series that predate treatment intensification.

Randomized trials of prostate radiotherapy (RT) established that treatment of the primary tumor can be clinically relevant in low-volume de novo metastatic disease. STAMPEDE demonstrated an overall survival (OS) benefit in the low-volume subgroup, while PEACE-1 showed that prostate RT added to intensified systemic therapy improved radiographic progression-free survival (PFS) in low-volume disease and reduced serious genitourinary events, without a demonstrated OS improvement [8,9]. These trials support controlling the primary tumor but do not establish equivalence between RT and radical prostatectomy (RP), which differ in selection, timing, tissue removal, nodal staging, morbidity, and recovery.

The question addressed here is not whether robot-assisted radical prostatectomy (RARP) can technically be performed in OMPC, but whether cytoreductive prostatectomy offers enough oncologic or local-control benefit to justify its risks in the era of intensified systemic therapy and molecular imaging. RARP offers well-established perioperative advantages over open surgery in conventional indications, but those advantages must not be confused with evidence that removal of the primary tumor improves survival [10,11,12,13]. Two levels of evidence must therefore be kept separate throughout this review. The first concerns RP as an oncologic strategy: all of the highest-level evidence discussed—RAMPP, the trials by Dai and by Chapin and colleagues, TRoMbone and IP2-ATLANTA—evaluated prostatectomy or local treatment irrespective of surgical approach, and none was designed to test, or establishes, a robot-specific oncologic effect. The second concerns RARP as the surgical platform through which cytoreductive prostatectomy is delivered in most contemporary centers, with the perioperative, functional, and technical considerations that follow from that choice. Throughout the text, “cytoreductive prostatectomy” therefore denotes the oncologic strategy, whatever the approach, and “cytoreductive RARP” is used only when the robotic platform itself—its perioperative profile, technique, or planning—is the subject.

This review develops a framework organized around that distinction: the clinical states in which surgery has been studied, the evidence hierarchy from registries to randomized trials, the perioperative and functional consequences of the robotic approach, a selection framework, and integration with systemic therapy, RT, and metastasis-directed therapy (MDT)—set against a guideline position that still restricts surgery for M1 disease to clinical trials [7].

## 2. Evidence Acquisition

This article is a narrative review. We searched PubMed/MEDLINE from database inception to 1 July 2026 for English-language studies addressing treatment of the primary tumor in oligometastatic and low-volume metastatic prostate cancer. The strategy combined controlled-vocabulary and free-text terms for oligometastatic or low-volume metastatic prostate cancer with terms for radical prostatectomy, robot-assisted prostatectomy, cytoreductive surgery, primary-tumor treatment, PSMA PET, systemic-therapy intensification, metastasis-directed therapy, and quality of life. The complete search strings, limits, and dates are provided in Supplementary Table S1.

We additionally hand-searched the reference lists of key systematic reviews and primary studies, reviewed the 2026 European Association of Urology (EAU) guideline on prostate cancer, and searched ClinicalTrials.gov, the ISRCTN registry, and the EU Clinical Trials Register (last searched 1 July 2026) for ongoing, completed, and unpublished trials of surgery for the primary tumor in metastatic prostate cancer.

Evidence was prioritized according to its ability to answer a surgical question. Randomized and prospective comparative trials were given the greatest weight, followed by prospective cohorts, contemporary PSMA PET-staged series, comparative retrospective studies, and registry analyses, the last interpreted as hypothesis-generating. Evidence on systemic therapy, prostate RT, and MDT was retained only where it defined the selection, sequencing, alternatives, or limitations of surgery. Where sources disagreed, we retained both and addressed the discrepancy explicitly, including the composition of overlapping meta-analyses. Studies were appraised narratively, with attention to selection bias, imaging era, and the systemic-therapy background against which surgery was assessed. Because this is a narrative rather than a systematic review, no formal risk-of-bias scoring or quantitative synthesis was performed and no protocol was registered.

## 3. Defining the Disease State Relevant to Surgery

### 3.1. Oligometastatic Taxonomy

A simple lesion count is clinically convenient but biologically incomplete. The ESTRO/EORTC framework distinguishes de novo oligometastatic disease, oligorecurrence after definitive treatment, oligoprogression during otherwise controlled systemic therapy, and other states defined by the timing and behavior of metastases [3]. For cytoreductive prostatectomy the relevant population is de novo synchronous mHSPC with a resectable primary tumor, because the prostate remains untreated and may contribute to later local morbidity or systemic burden. Metachronous disease after prostatectomy is by definition not a setting for primary RP, and after prior RT it becomes a salvage question with a different risk profile.

“Oligometastatic” and “low-volume” should not be used interchangeably. CHAARTED low-volume disease is defined by the absence of visceral metastases and the absence of high-volume bone disease (four or more bone metastases with at least one beyond the vertebral bodies and pelvis), while many OMPC studies use a numerical threshold of one to three or one to five lesions. A man with four axial bone metastases may be low volume by CHAARTED yet exceed a surgical trial threshold, while a few visceral lesions may be numerically oligometastatic but biologically unfavorable. Studies should report lesion count, distribution, synchronous versus metachronous presentation, and the burden definition used (Supplementary Table S2). In this review, “low-volume” refers to CHAARTED-type criteria as used in STAMPEDE and PEACE-1 (STAMPEDE’s own term, “low burden”, is retained only when that trial is quoted), and “oligometastatic” refers to lesion-count definitions, which vary between studies and are specified where each study is cited.

### 3.2. Imaging-Era Dependence and Stage Migration

The apparent oligometastatic state is inseparable from the imaging method used to define it. Most pivotal systemic and primary-RT trials relied on computed tomography and bone scintigraphy. PSMA PET/CT is more accurate for nodal and distant staging in high-risk prostate cancer (PCa) and frequently identifies additional lesions, producing stage migration from apparently localized or low-volume disease to molecularly metastatic disease [14]. This cuts both ways: molecular imaging may exclude men with unrecognized widespread disease from surgery but also identifies very small-volume metastases whose natural history and response to local treatment are unknown.

A PSMA PET-defined patient with two small nodal lesions is not comparable with a conventionally staged patient with two bone metastases, so cohorts should report both molecular and conventional burden, the distribution of PSMA-avid disease, and whether imaging altered the planned nodal dissection. Without this granularity, apparent gains across eras may reflect migration to more favorable disease rather than treatment effect.

### 3.3. Biological and Clinical Rationale for Removing the Primary Tumor

The biological rationale for cytoreduction includes reduction in total tumor burden, removal of a possible source of circulating tumor cells (CTCs), interruption of tumor-derived signals that support metastatic niches, and modification of systemic immune or inflammatory pathways. Preclinical and translational studies support the plausibility of these mechanisms, and perioperative changes in CTCs have been described in patients undergoing cytoreductive prostatectomy [15,16,17,18,19]. However, biological plausibility is not a surrogate for clinical benefit. A reduction in tumor-cell shedding or a postoperative biomarker change does not prove delayed castration resistance, improved quality of life, or longer survival.

The most immediate clinical rationale may be local rather than systemic. Removing the prostate can prevent or reduce bladder-outlet obstruction, hematuria, ureteric obstruction, catheter dependence, transurethral procedures, and other local interventions. It also provides complete pathologic staging, margin information, and nodal assessment. These benefits are meaningful but must be balanced against incontinence, lymphocele, anastomotic complications, thromboembolic events, sexual dysfunction, and the possibility of delaying systemic therapy.

### 3.4. Prostate Radiotherapy as the Reference Local Therapy

STAMPEDE established prostate RT as a standard component of treatment for selected men with newly diagnosed low-volume metastatic disease [8]. PEACE-1 extended the context to an era of intensified systemic therapy: in the low-volume population receiving abiraterone, prostate RT prolonged radiographic PFS and reduced serious genitourinary events but did not improve OS [9]. The current EAU guideline strongly recommends prostate RT for patients presenting with low-volume M1 disease by CHAARTED criteria, while strongly advising against combining ADT with surgery outside clinical trials [7]. The peer-reviewed STOPCAP aggregate-data meta-analysis of STAMPEDE and HORRAD confined the survival benefit to men with fewer than five bone metastases, in whom 3-year survival improved by approximately 7% [20]. An updated STOPCAP individual-participant-data analysis incorporating PEACE-1, presented in abstract form at ESMO 2025 and updated at APCCC 2026 but not yet published in full at the time of writing, similarly confined the benefit to men with fewer than five bone metastases, with an absolute 5-year survival gain of approximately 5–6% [21]; these estimates are conference-reported and should be regarded as preliminary until the full publication is available. This is precisely the population in which cytoreductive surgery is also proposed, so any surgical strategy must be judged against an active comparator with a demonstrated survival effect rather than against systemic therapy alone.

## 4. Evidence for Cytoreductive Prostatectomy and RARP

### 4.1. Registry Analyses: Hypothesis Generation, Not Causal Evidence

The first major clinical signals arose from cancer registries. Analyses of the Munich Cancer Registry, the Surveillance, Epidemiology, and End Results (SEER) program, and SEER-Medicare associated treatment of the primary tumor with improved cancer-specific or overall survival in selected men with metastatic PCa [22,23,24,25]. They challenged the assumption that local treatment had no role once metastases were present.

They are, however, highly vulnerable to immortal-time and selection bias—men chosen for surgery are younger, fitter, lower-burden, and treated at specialist centers.

### 4.2. Comparative and RARP-Specific Observational Studies

Focused cohorts report feasibility and favorable outcomes in selected responders with low-volume skeletal metastases [26], and better progression and cancer-specific outcomes with RARP plus systemic therapy than ADT alone in 79 men with bone oligometastases [27]. A prospective case–control study provides the counterweight, showing no clear survival advantage though fewer locoregional complications [28].

RARP-specific evidence remains observational: propensity-matched data show the operation can be completed in OMPC with short-term outcomes comparable to localized disease [29]. This supports technical feasibility but not oncologic superiority over systemic therapy or RT.

### 4.3. Prospective Cohorts and Contemporary PSMA PET-Staged Evidence

The LoMP program prospectively evaluated local treatment in metastatic PCa and demonstrated that cytoreductive prostatectomy can be integrated into real-world multimodal pathways [30,31]. Its findings on local control, functional recovery, and subsequent treatment are more informative than registry coding, but treatment allocation was not randomized. Patients and clinicians chose between surgery, RT, or no local treatment, leaving substantial confounding by preference, resectability, symptoms, disease burden, and perceived prognosis.

The multicenter European PSMA PET-staged cohort by Rajwa and colleagues represents one of the most contemporary surgical datasets. Among 116 patients treated at 13 centers, most had PSMA-defined bone metastases, systemic therapy and MDT were common, any perioperative complication occurred in approximately one third, major complications were uncommon and 1-year continence was reported in the majority. Intermediate-term castration-resistant-free and overall survival estimates were encouraging [32,33]. The absence of a nonsurgical comparator, short follow-up, expert-center setting, and multimodal treatment prevents attribution of these outcomes to prostatectomy.

### 4.4. Prospective Feasibility and Randomized Evidence

TRoMbone was a crucial methodological step. It randomized men with synchronous bone-oligometastatic PCa to standard systemic therapy with or without RP and demonstrated that recruitment, randomization, and surgery were feasible in specialist centers [34]. The trial was deliberately not powered for survival, and its main contribution is proof that a definitive randomized surgical study is possible.

The phase II trial by Dai and colleagues randomized 200 patients with newly diagnosed OMPC (five or fewer bone or extrapelvic nodal metastases, no visceral disease) to ADT alone or ADT plus radical local treatment, 100 per arm. In the local-treatment arm, 96 of 100 patients received local therapy (85 RP and 11 prostate RT), and 17 of 100 control patients eventually received local treatment; all were analyzed by intention to treat. At a median follow-up of 48 months, median radiographic PFS was not reached with local treatment versus 40 months without it (hazard ratio 0.43, 95% confidence interval 0.27–0.70), and the 3-year OS rate was 88% versus 70% (hazard ratio 0.44, 95% confidence interval 0.24–0.81) [35]. Because surgery and RT were combined within the experimental strategy, the trial supports treatment of the primary tumor but cannot isolate a prostatectomy effect, or the contribution of local therapy relative to differences in subsequent systemic management.

A randomized phase II trial by Chapin and colleagues reached the opposite conclusion. Of 180 men with de novo M1 disease enrolled and treated with 6 months of best systemic therapy, 26 (14%) progressed during induction and 35 (19%) were not randomized because of patient or clinician preference; 119 (66%) were randomized to continue systemic therapy alone (*n* = 59) or to add definitive local treatment (*n* = 60), of whom 45 (75%) underwent surgery, 13 (22%) received RT, and 2 (3%) received neither. Median PFS was 17.9 months (95% confidence interval [CI] 11.7–36.4) with systemic therapy alone and 14.8 months (95% CI 11.4–42.9) with added local treatment; the reported hazard ratio (HR) for the local-treatment arm relative to systemic therapy alone was 0.89 (95% CI 0.59–1.34; *p* = 0.6). The apparent discordance between the medians and the HR reflects wide, overlapping confidence intervals and non-proportional, late-separating survival curves rather than an error, and the result should be read as showing no demonstrable PFS difference in either direction. No OS difference was observed at a median follow-up of 66 months, and grade 3 toxicity occurred in 4 of 60 patients (6.7%) in the local-therapy arm and in none of the 59 control patients [36]. Three features of this trial bear directly on patient selection for surgery. First, randomization took place only after a 6-month period of best systemic therapy, so the trial tested local therapy in a population already filtered for initial systemic response. Second, best systemic therapy consisted of ADT in all 119 randomized patients, with docetaxel in 37 and an androgen receptor pathway inhibitor (ARPI) in only 9, so the control arm again predates contemporary intensification. Third, an aggressive-variant molecular signature, defined by abnormality of at least two of p53, RB1, and PTEN at baseline or at 6 months, was associated with worse PFS (HR 1.74, 95% CI 1.02–2.98), while CHAARTED high-volume disease predicted worse OS (HR 1.84, 95% CI 1.06–3.19), with a similar but nonsignificant trend for cT3b–T4 disease. The design therefore illustrates both the value of a systemic-therapy induction period as a biological filter and the possibility that, once that filter is applied, adding local treatment yields no measurable progression benefit.

RAMPP (NCT02454543) is the first completed randomized efficacy trial specifically evaluating RP plus best systemic therapy against best systemic therapy alone. Between May 2015 and December 2018, 132 patients with one to five bone metastases, with or without nodal involvement, on conventional or PET imaging were randomized (66 per arm). After a median follow-up of 5 years, cancer-specific death had occurred in 21 of 132 patients (16%); the 5-year cumulative incidence of cancer-specific mortality (CSM) was 13% with RP plus systemic therapy and 23% with systemic therapy alone (*p* = 0.037), corresponding to an HR of 0.39 (95% CI 0.16–0.98; *p* = 0.045) in competing-risk regression. The 5-year cumulative incidence of clinical progression was almost identical (59% versus 60%), the 5-year OS rate was 81% versus 74% and not statistically significant, and Clavien–Dindo grade ≥III surgery-related complications occurred in 9 of 66 patients (14%) in the surgical arm [37]. Accrual stopped at 132 of 452 planned patients because clinical practice changed during recruitment—in particular after STAMPEDE established prostate RT for low-volume disease—and because recruitment was slow.

RAMPP materially changes the evidence hierarchy: the field can no longer be described as having feasibility evidence alone, because a randomized CSM signal for prostatectomy now exists. Its limitations, however, prevent a standard-of-care conclusion, and the accompanying editorial by Sooriakumaran, Bryant and Hamdy sets them out explicitly [38]. First, early closure and only 21 cancer-specific deaths make the estimate fragile, and the upper confidence limit of the HR approaches unity. Second, CSM is vulnerable to misattribution of the cause of death, whereas the OS difference was not significant and the broader clinical-progression endpoint was unchanged. Third, initial best systemic therapy was ADT alone in the large majority of patients; accrual predates ARPI-based intensification, and the editorial’s central argument is that a trial run against an outdated control cannot inform present-day practice. Fourth, 14% major surgical morbidity is a real cost for an uncertain gain. The editorial accordingly characterizes RAMPP as a signal requiring confirmation rather than a basis for changing practice and calls for new randomized evidence before RP can be recommended for low-volume metastatic disease outside clinical trials—the position adopted in this review.

The size of the systemic-therapy gap deserves quantification, because its direction is not intuitive. Adding abiraterone to ADT reduced the hazard of death by roughly one third in mHSPC: the HR for OS was 0.63 (95% CI 0.52–0.76) in the STAMPEDE abiraterone comparison and 0.66 (95% CI 0.56–0.78) in the final analysis of LATITUDE, where median OS improved from 36.5 to 53.3 months [39,40]; comparable gains have since been shown with other ARPIs and with triplet therapy [7]. The RAMPP control arm, treated largely with ADT alone, therefore probably experienced substantially higher CSM than a contemporary control arm would. Two consequences follow. When the control arm does poorly, a fixed absolute benefit of local treatment is easier to detect and appears larger in relative terms, so the RAMPP HR of 0.39 may overstate the contribution surgery would make on top of modern intensification—the same mechanism commonly invoked to explain why PEACE-1 found no OS benefit from prostate RT once abiraterone was added [9]. Conversely, it is also possible that intensified systemic therapy leaves a smaller residual burden in which local treatment is more often curative, as the persistence of viable tumor in IP2-ATLANTA (below) might suggest. Which effect dominates is unknown, and that uncertainty is itself an argument for trials rather than adoption. The relevant question today is not whether prostatectomy adds benefit to ADT-based therapy, but whether it adds clinically meaningful benefit beyond modern systemic intensification, prostate RT, and MDT—a question RAMPP was neither designed nor powered to answer [37,38].

The randomized results therefore do not point in the same direction. RAMPP reports a CSM advantage for prostatectomy in a bone-metastatic population selected on lesion count; the trial by Chapin and colleagues, in a population filtered by response to 6 months of systemic therapy and treated predominantly with surgery, shows no progression or survival benefit from local treatment. Randomized evidence for surgical cytoreduction is therefore mixed rather than uniformly encouraging, and the discrepancy may reflect differences in selection, in the endpoint chosen, and in how much systemic therapy preceded randomization.

The IP2-ATLANTA internal pilot (NCT03763253) adds a third randomized perspective. Of 108 eligible men, 81 (75%) were randomized to standard of care (*n* = 28), minimally invasive ablative therapy (*n* = 27), or radical treatment (*n* = 26). In the radical arm, 19 of 26 (73%) received the allocated intervention—cytoreductive RP in 5 (19%) and prostate RT in 14 (54%)—whereas the remaining 7 (27%) did not, because of withdrawal of consent for the intervention, unfitness for treatment, or clinical deterioration, two patients having died of prostate cancer before the planned intervention. Grade ≥3 adverse events were reported in 5 of 28 (18%) control, 2 of 26 (7%) ablation-arm, and 4 of 26 (15%) radical-arm patients, so intervention was not more toxic than standard care. More instructive for surgical decision-making, a complete pathological response after systemic therapy was documented in only 6 of 53 intervention-arm patients (11%; 95% CI 4.3–23.0), assessed on repeat biopsy or whole-mount prostatectomy specimens in the 32 patients with tissue available, 26 of whom (81%) had residual adenocarcinoma [41]. That observation is the strongest available argument that the primary tumor is not reliably sterilized by contemporary doublet systemic therapy, and it provides a coherent biological rationale for local therapy. It does not, however, establish that removing residual disease alters survival, nor does it favor surgery over RT, given that most radical-arm patients were irradiated rather than operated on.

### 4.5. Systematic Reviews and Certainty of Evidence

Systematic reviews consistently describe favorable survival associations after cytoreductive prostatectomy, but their conclusions are constrained by the quality of the underlying studies, and they should not be read as independent confirmations of one another: they draw on a small and heavily overlapping pool of primary studies dominated by SEER and Munich registry analyses and by the same institutional cohorts, and two of the syntheses were produced by the same author group [42,43,44,45,46]. The 2025 meta-analysis by Rajan and colleagues included eight studies and 611 patients. It reported associations with improved OS and cancer-specific survival, but not statistically significant improvements in PFS or castration-resistant-free survival; fewer local events were also reported. The certainty of survival evidence was judged very low and that for local events low [43].

An earlier systematic review of 11 studies and 511 patients reached a more restrictive conclusion. It reported acceptable morbidity in selected patients, with positive surgical margins in 59% and Clavien–Dindo grade 3 or higher complications ranging from 0% to 21%, but judged that the absence of a consensual definition of the oligometastatic state and the low level of available evidence precluded practical recommendations, concluding that surgery for metastatic disease had no role outside a clinical trial [47].

A 2026 umbrella review placed these syntheses within a formal hierarchy of credibility. Across 21 meta-analyses enrolling more than 160,000 patients, prostate RT in low-volume mHSPC was supported by highly suggestive evidence of an OS benefit, whereas cytoreductive prostatectomy reached only suggestive credibility for OS and cancer-specific survival and demonstrated no superiority over RT. Local treatment approximately halved the risk of symptomatic pelvic events [48]. For surgical cytoreduction the implication is twofold: the direction of effect is consistent across syntheses, but because those syntheses largely share the same primary studies, consistency should not be mistaken for independent replication, and the credibility of the surgical evidence remains a tier below that of prostate RT.

These findings illustrate why pooled effect sizes should not be read as definitive treatment effects. Combining small observational studies can increase precision without removing confounding, and overlapping cohorts can create apparent replication. Variability in lesion thresholds, conventional versus PSMA imaging, systemic regimens, use of MDT, surgical approach, surgeon volume, endpoint definitions, and follow-up further limits generalizability. The most defensible synthesis is that prostatectomy is feasible, may reduce local morbidity, and now has an initial randomized CSM signal that is not corroborated by another randomized trial, while definitive OS benefit remains unproven. We summarize the key evidence informing cytoreductive prostatectomy and RARP in Table 1.

## 5. RARP as the Surgical Platform

### 5.1. Why a Robotic Approach Is Attractive

The considerations in this section concern the surgical platform, not oncologic efficacy: no comparative study in OMPC has tested robotic against open or laparoscopic prostatectomy, and the perioperative advantages summarized below are extrapolated from localized and high-risk disease. RARP has become the dominant approach to RP in many high-volume systems. In localized and high-risk disease, it is associated with lower blood loss, lower transfusion rates, shorter hospitalization, and faster early recovery than historical open surgery, with broadly comparable short-term oncologic outcomes [10,11,12,13]. These properties are particularly relevant in OMPC because patients require ongoing systemic therapy and may also require prostate-bed, pelvic, or metastatic-site RT. A shorter recovery could reduce interruption of the multimodal pathway.

Robotic magnification and instrument articulation may facilitate dissection around a bulky primary tumor, bladder neck reconstruction, ureteric identification, and extended pelvic lymph-node dissection (ePLND) [49,50,51,52]. However, apparent technical precision should not be equated with oncologic completeness. Positive margins may remain frequent in advanced primary tumors, and extended nodal dissection adds operative time and lymphatic morbidity. RARP should be performed by surgeons with high-risk and locally advanced experience, not merely routine robotic-volume credentials.

### 5.2. Preoperative Planning

Preoperative planning must address four domains: systemic disease, local resectability, pelvic nodal disease, and the downstream treatment plan. High-quality prostate magnetic resonance imaging can define extracapsular extension, seminal-vesicle involvement, bladder-neck or apical disease, and potential rectal involvement. PSMA PET/CT should characterize nodal and distant distribution, while conventional imaging remains relevant when applying evidence or guidelines derived from conventionally staged trials [4,14]. The surgeon should anticipate whether negative margins are plausible and whether surgery will meaningfully reduce local morbidity.

The systemic plan must be agreed beforehand: induction agent and response interval, the threshold for cancelling surgery because of progression, the timing of postoperative systemic therapy, and any planned MDT or prostate-bed RT. RARP should not proceed without a credible plan for the metastatic component; the operation is one element of a multimodal sequence, not a standalone curative procedure.

### 5.3. Operative Principles and Technical Challenges

The operation generally follows high-risk RARP principles, but the balance between functional preservation and oncologic control is shifted. Nerve sparing may be inappropriate in extensive extracapsular disease, and wide apical or posterolateral excision may be required. Bladder-neck involvement can necessitate reconstruction, while bulky nodal disease may require complex vascular or ureteric dissection. The extent of ePLND should be defined by oncologic rationale and imaging rather than by the assumption that removal of all PSMA-avid nodes is achievable or curative.

Prior systemic therapy may reduce tumor volume but can alter tissue planes; evidence that it reliably increases operative difficulty is inconsistent, though fibrosis and loss of tissue contrast may be encountered. Reporting should be standardized to include operative time, blood loss, transfusion, nodal yield, positive margins, anastomotic complications, lymphocele, readmission, and time to resumption of systemic therapy.

### 5.4. Perioperative Morbidity

Perioperative results vary substantially. In a multicenter analysis of 106 men undergoing RP for metastatic disease, most experienced no complication, but positive margins were common and lymphocele and wound infection were reported [53]. TRoMbone demonstrated acceptable feasibility in selected patients, whereas the phase I study by Yuh and colleagues reported approximately one-third overall 90-day morbidity, 6.25% major morbidity, and one postoperative death [34,54]. These findings demonstrate that cytoreductive prostatectomy is not simply routine RARP performed in a patient with a few additional lesions.

The variability reflects selection, primary-tumor extent, surgeon experience, and differing definitions: a low complication rate in a high-volume center should not be extrapolated to lower-volume practice. Future trials need surgical credentialing, central complication review, and transparent reporting of patients not operated after randomization.

### 5.5. Functional Outcomes and Quality of Life

Continence is one of the most important trade-offs. A multi-institutional comparison found longer surgery, greater blood loss, and lower 1-year pad-free continence after cytoreductive prostatectomy than after RP for localized disease [55]. More favorable continence estimates have been reported in contemporary PSMA PET-staged cohorts, and a selected health-related quality-of-life (HRQoL) study did not demonstrate a significant overall deterioration after cytoreductive RP [32,56]. Small single-center series report similarly high figures, including 1-year continence in 10 of 12 men treated with neoadjuvant ADT followed by open cytoreductive prostatectomy, but such cohorts are highly selected and report short follow-up [57]. Different continence definitions, baseline function, nerve-sparing rates, ADT exposure, and expertise explain some of the variation.

Sexual outcomes are even harder to interpret. Many patients receive long-term ADT, which impairs libido and erectile function independently of surgery. Locally advanced disease often precludes nerve sparing, and recovery may be less important to some patients than freedom from catheters or local symptoms. Nevertheless, sexual function and bother should be measured, not assumed to be irrelevant. Patient-reported outcomes should be collected longitudinally from baseline and analyzed alongside systemic-treatment toxicity.

### 5.6. Local Control, Pathology, and Postoperative Information

Local control is the most plausible near-term benefit of prostatectomy. Prospective and registry studies suggest fewer locoregional events or subsequent local interventions after surgery, even when survival is not clearly improved [28,31,43]. A dedicated meta-analysis quantifies this more precisely and is the strongest quantitative argument for surgery over radiotherapy on a non-survival endpoint: across six studies, adding local treatment halved overall local events (relative risk 0.50, 95% confidence interval 0.28–0.88), but the effect was concentrated in the surgical subgroup, where prostatectomy reduced overall local events (relative risk 0.24, 95% confidence interval 0.11–0.52) and local events requiring surgical intervention (relative risk 0.08, 95% confidence interval 0.03–0.25). Prostate RT did not significantly reduce overall local events, although it did reduce those requiring intervention [58]. This is a durable and clinically meaningful advantage, but it is a morbidity endpoint rather than a survival one, and it must be weighed against the perioperative and continence costs described above. Complete pathology also identifies grade, stage, margins, nodal burden, treatment effect, intraductal or cribriform architecture, and other adverse features that may influence subsequent therapy. Yet more information is not itself a clinical benefit unless it leads to an intervention that improves outcomes.

Pathologic response after induction therapy is a promising prognostic marker. A recent retrospective surgical cohort associated greater treatment effect with more favorable outcomes [59]. Because pathologic response is known only after the operation, it cannot independently select patients before surgery. Its future value may lie in response-adapted postoperative strategies or as an intermediate endpoint in neoadjuvant trials. Prospective validation is needed to distinguish a prognostic marker from a predictor of benefit from surgery.

## 6. Selecting Patients for Cytoreductive Prostatectomy

### 6.1. Disease-Related Factors

The selection criteria discussed here derive from the populations enrolled in studies of cytoreductive prostatectomy irrespective of surgical approach; they are not RARP-specific, and they describe an investigational rather than a validated pathway. The most defensible surgical candidate has de novo, non-visceral, low-volume mHSPC; a limited number of bone or nonregional nodal metastases; a resectable primary tumor; and no evidence of rapid systemic progression. This reflects the populations enrolled in surgical studies, not a validated predictive rule; visceral metastases, extensive bone disease, bulky unresectable pelvic disease, or progression despite systemic therapy are unlikely to justify surgical risk.

Distribution may matter as much as lesion count: nodal-only disease differs biologically from bone metastases, and nonregional nodes raise questions about the extent and value of dissection. Sites that cannot be safely treated with MDT do not preclude surgery but weaken the total-disease-control rationale. We provide a practical, explicitly investigational selection framework for cytoreductive prostatectomy in Table 2.

### 6.2. Patient-Related and Surgical Factors

Patients should have Eastern Cooperative Oncology Group (ECOG) performance status 0–1, sufficient life expectancy, acceptable anesthetic risk, and the physiological reserve to recover without compromising systemic treatment. Age alone should not be an absolute exclusion, but comorbidity and frailty increase perioperative risk and competing mortality [62,63]. Baseline urinary, continence, and erectile function, prior pelvic surgery, and patient priorities should be documented beforehand.

Resectability must be judged by an experienced robotic surgeon, since a technically possible operation is not necessarily an oncologically sensible one: rectal fixation, unreconstructable bladder-neck involvement, or disproportionate vascular risk may favor RT. Center and surgeon volume are themselves selection criteria.

### 6.3. Treatment Response and Biomarkers

A deep prostate-specific antigen (PSA) response to initial systemic therapy is frequently used as a marker of favorable biology, but no threshold is validated to predict benefit from prostatectomy. A strong response may identify disease controlled enough to tolerate local consolidation but equally may identify men already doing well on systemic therapy, shrinking the incremental value of surgery. Lack of response should generally prompt reconsideration.

Quantitative PSMA PET metrics may refine selection. In a retrospective study of low-volume mHSPC, lower whole-body PSMA-derived tumor volume (PSMA-TV) and absence of nonregional nodal disease were associated with more favorable PFS among patients considered for cytoreductive RP [60]. Preoperative PSMA-positive pelvic nodes have also been associated with PSA persistence after RP [61]. These findings are prognostic and hypothesis-generating and should not become rigid eligibility thresholds without prospective validation and treatment-interaction testing.

### 6.4. Multidisciplinary and Preference-Sensitive Selection

Every candidate should be discussed by a multidisciplinary prostate-cancer team including urology, medical oncology, radiation oncology, nuclear medicine or radiology, and pathology [64]. The team should weigh at least three strategies—systemic therapy alone, systemic therapy plus guideline-supported prostate RT, and an investigational surgical pathway with or without MDT—and document the intended benefit and the plan if disease progresses before surgery.

Patient preferences can materially alter the decision. In IP5-MATTER, patients valued survival and progression-free intervals but also placed substantial weight on urinary function, fatigue, and treatment burden; prostatectomy was less attractive unless combined with MDT [65]. Counseling should convey the possibility of RT after surgery, the likelihood of continuing systemic therapy, and the fact that a technically successful RARP may not lengthen survival.

## 7. Integrating RARP into Multimodal Treatment

### 7.1. Systemic Therapy Is the Backbone

Cytoreductive prostatectomy, by whatever approach, must be embedded within guideline-concordant systemic treatment. Contemporary EAU recommendations favor ADT combined with an ARPI and reserve docetaxel for appropriate intensified combinations in fit patients [7]. Surgical cohorts spanning older eras may therefore overestimate the incremental value of local treatment. Trials should standardize or stratify systemic regimens and report adherence, duration, and subsequent therapy.

The timing of local treatment relative to systemic intensification remains unresolved. Induction treats occult disease immediately and allows biology to declare itself, but alters tissue planes, creates a selection period in which only responders reach surgery, and complicates intention-to-treat interpretation. Upfront surgery gives immediate local control and pathology but may delay systemic treatment. No randomized trial has compared these sequences in OMPC.

### 7.2. Surgery Versus Prostate Radiotherapy

For a patient meeting the low-volume criteria used in RT trials, prostate RT is the evidence-based local standard [7,8,9]. Surgery should not be presented as an equivalent established alternative; the 2026 umbrella review found no superiority of cytoreductive prostatectomy over prostate RT [48]. The literature is not unanimous: a meta-analysis by Cheng and colleagues of five comparative studies including 1425 patients reported better cancer-specific survival (HR 0.70, 95% CI 0.59–0.81) and OS (HR 0.80, 95% CI 0.77–0.82) after cytoreductive prostatectomy than after RT, with no difference in progression-free or castration-resistant-free survival [46]. Examining the composition of that synthesis explains most of the divergence. Its five studies were two SEER-based registry analyses with overlapping patients and calendar periods (Culp and colleagues, 245 RP versus 129 brachytherapy; Leyh-Bannurah and colleagues, 313 RP versus 161 RT) [23,25]; a Veterans Affairs cohort of men with metastatic castration-resistant disease in whom “prior local therapy” had been delivered years earlier for presumed localized cancer rather than as cytoreduction for de novo metastatic disease [66]; a small Chinese series in which the comparator for prostatectomy plus metastasis-directed RT was stereotactic RT to metastases alone, not prostate RT [67]; and the nonrandomized LoMP registry comparison (48 RP versus 26 RT), which the meta-analysis appraised as if it were a randomized trial [31]. Only the LoMP comparison is both prospective and specific to the clinical question, and the implausibly narrow confidence interval for OS reflects the weight of two registry analyses drawn from the same data source. The umbrella review, an analysis of published meta-analyses rather than of new data, graded the same body of registry-derived comparisons with formal credibility criteria that penalize small-study effects and heterogeneity, and reached a more conservative conclusion [48]. The two syntheses therefore disagree not because independent bodies of evidence conflict, but because one pools largely the same registry comparisons—in which surgical patients are systematically younger, fitter, and less locally advanced than irradiated patients, precisely the confounding by indication that a survival comparison cannot resolve—while the other discounts them. The disagreement should be read as evidence that the question is unsettled, not as support for surgery. A trial comparing surgery with systemic therapy alone may answer whether prostatectomy has activity, but it does not answer the modern clinical choice between RARP and RT. Comparative studies should measure not only survival but local events, urinary and bowel function, continence, sexual outcomes, treatment burden and need for salvage therapy.

The two may be complementary rather than exclusive, since some operated men need postoperative RT for adverse pathology, persistent PSA, or local recurrence—a possibility that raises treatment burden and should be discussed beforehand. Surgery after prior RT is salvage treatment with greater morbidity and should not be conflated with upfront cytoreductive prostatectomy. Protocols must define permitted postoperative RT so outcomes are attributed to the full pathway.

### 7.3. Metastasis-Directed Therapy

MDT, most commonly stereotactic body radiotherapy (SBRT), is increasingly used to control limited metastatic sites. Randomized phase II studies and meta-analyses show gains in progression-related endpoints, but OS evidence remains immature [68]. The 2026 WOLVERINE individual patient data meta-analysis of randomized trials found that MDT added to standard care improved PFS, radiographic PFS, and castration-resistant-free survival; the OS result did not reach conventional statistical significance [69]. Current EAU guidance restricts MDT in M1 disease to clinical trials or well-designed prospective cohorts [7].

The relevance to RARP is indirect: retrospective series combining cytoreductive prostatectomy with metastatic-site RT report encouraging outcomes, and total-eradication strategies show surgery can be incorporated into intensive multimodal care [67,70,71,72,73]. Such designs cannot separate the contributions of prostatectomy, MDT, systemic therapy, and favorable biology. An effective MDT strategy does not prove RARP adds value, and surgical trials should prespecify whether all visible metastases must be treated.

### 7.4. Neoadjuvant Intensification and Response-Adapted Surgery

Intense androgen receptor inhibition before RP produces pathologic responses in high-risk nonmetastatic PCa [74,75,76]. This supports biological activity and feasibility but cannot establish a survival benefit for neoadjuvant therapy followed by RARP in OMPC. Dedicated protocols—NEAR-TOP, CHAMPION, and a rezvilutamide-based study—are testing radiohormonal, maximal-cytoreductive, and intensified neoadjuvant strategies [77,78,79]. Until mature, these are trial designs, not evidence of efficacy.

A response-adapted model would begin with systemic therapy, reassess PSA and imaging at a predefined interval, and proceed to RARP only if disease remains limited and resectable. This is rational but enriches the surgical group for good responders, exaggerating apparent benefit in nonrandomized comparisons; randomization should therefore precede the response-selection period.

### 7.5. A Pragmatic Clinical Pathway

**Figure 1:**
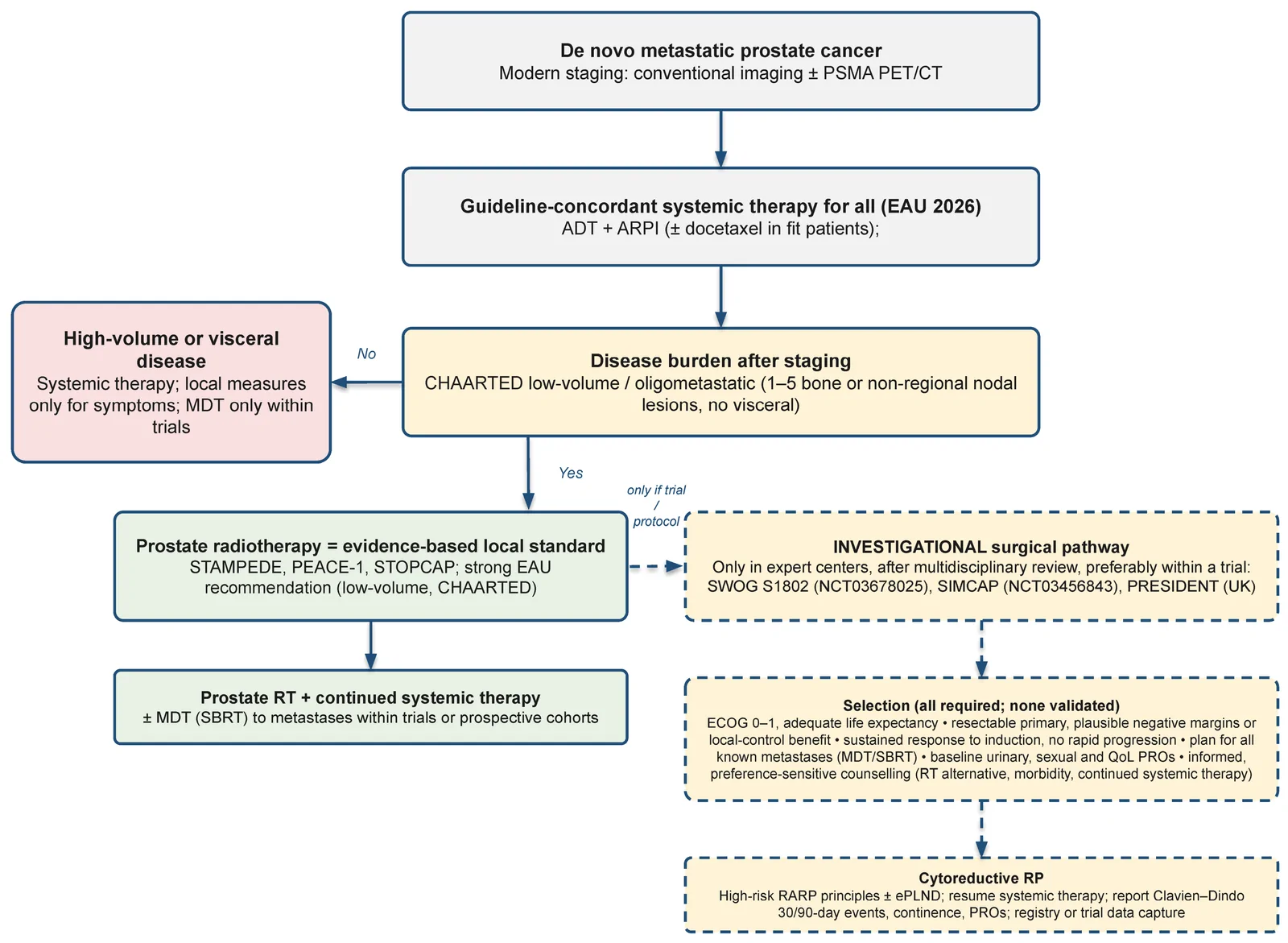
Evidence-based, explicitly investigational pathway locating cytoreductive prostatectomy relative to systemic therapy, prostate radiotherapy, metastasis-directed therapy, patient selection, and ongoing trials. Solid boxes indicate guideline-supported steps; the dashed box indicates the investigational surgical pathway, which current EAU guidance restricts to clinical trials [7]. ADT, androgen deprivation therapy; ARPI, androgen receptor pathway inhibitor; CI, confidence interval; CSM, cancer-specific mortality; ECOG, Eastern Cooperative Oncology Group; ePLND, extended pelvic lymph-node dissection; HR, hazard ratio; MDT, metastasis-directed therapy; OS, overall survival; PFS, progression-free survival; PROs, patient-reported outcomes; PSMA PET/CT, prostate-specific membrane antigen positron emission tomography/computed tomography; QoL, quality of life; RARP, robot-assisted radical prostatectomy; RP, radical prostatectomy; RT, radiotherapy; SBRT, stereotactic body radiotherapy.

Outside a trial, the safest evidence-conscious pathway (Figure 1) begins with guideline-concordant systemic therapy and modern staging. Patients with de novo low-volume disease should be offered prostate RT according to current guidance [7]. Cytoreductive prostatectomy, usually delivered as RARP, may be discussed only with fully informed patients at expert centers where there is a compelling local-control rationale and, preferably, an open trial or prospective protocol, and it should be documented as preference-sensitive, evidence-limited and investigational.

Before proceeding, the team should confirm: (1) low-volume, non-visceral disease; (2) no rapid progression during systemic therapy; (3) a resectable primary tumor; (4) acceptable perioperative risk; (5) a plan for all known metastatic disease; (6) a postoperative systemic-treatment strategy; and (7) baseline and planned follow-up assessment of urinary, sexual, and quality-of-life outcomes.

## 8. Current Guideline Position and Clinical Implications

The 2026 EAU guideline provides the clearest current practice boundary. It strongly recommends combination systemic therapy for fit patients with M1 disease, strongly recommends prostate RT for patients presenting with low-volume disease by CHAARTED criteria, strongly advises against ADT combined with surgery outside clinical trials, and restricts MDT to trials or well-designed prospective cohorts [7]. Thus, even after RAMPP, cytoreductive surgery remains investigational rather than guideline-endorsed care, a position coherent with an evidence hierarchy in which RT rests on larger phase III programs and surgery on a single underpowered trial with a nonsignificant OS result. The surgical community should avoid responding to a promising CSM signal by broad uncontrolled adoption, because doing so would make definitive trials harder to complete and expose patients to morbidity without a reliable estimate of net benefit.

## 9. Research Priorities and Future Directions

### 9.1. Trial Design

The cleanest design is systemic therapy with versus without RP in a prospectively defined low-volume population, though a contemporary trial may need prostate RT as an active comparator or a multi-arm platform. Stratification should cover imaging modality, nodal versus bone disease, burden, systemic regimen, induction response, and planned MDT.

Recruitment is a major challenge. A feasibility study randomized only 43 of 170 eligible patients: unresectable disease and inadequate surgical fitness were the leading causes of ineligibility, while patient refusal and enrolment in competing studies accounted for most non-randomization among eligible men [80]. Trials must engage patients early and describe surgery and RT even-handedly.

Three randomized trials in progress should resolve part of this. SWOG S1802 (NCT03678025), a phase III trial open since September 2018 with an estimated enrollment of 1273 men, randomizes systemic therapy with or without definitive treatment of the primary tumor (surgery or RT), is powered for OS with a prespecified surgical-intent subset, and was still recruiting at the time of revision, with primary completion estimated for 2028 [81]. SIMCAP (NCT03456843), a phase 2.5 multi-institutional trial randomizing cytoreductive prostatectomy plus best systemic therapy against best systemic therapy alone in newly diagnosed metastatic disease, with an estimated 190 participants, is active but no longer recruiting, with primary completion estimated for March 2027 [82]. PRESIDENT, a multicentre UK randomized trial funded by the National Institute for Health and Care Research Health Technology Assessment programme, plans to randomize 749 men with low-volume mHSPC defined on PSMA PET/CT (four or fewer skeletal or extra-pelvic nodal metastases) to RP plus contemporary systemic therapy (ADT plus an ARPI, with or without docetaxel and RT) or to systemic therapy alone, with a composite primary endpoint of deterioration-free survival that incorporates health-related quality of life; the design was presented in 2026 and the trial is in its internal pilot phase, with recruitment planned across 25–26 sites [83]. Only SIMCAP and PRESIDENT isolate prostatectomy; because surgery and RT share an arm in SWOG S1802, that trial will quantify local therapy rather than surgery. Until these mature, the surgical question remains open, and the interpretation of any single completed trial should be provisional.

### 9.2. Endpoint Selection

OS remains definitive but demands large samples and long follow-up. CSM, radiographic PFS, and time to castration resistance are relevant but sensitive to surveillance and subsequent therapy. Local event-free survival should be prospectively defined using catheterization, obstruction, hematuria, transurethral or ureteric intervention, palliative pelvic RT, and hospitalization rather than retrospective coding.

Surgical and patient-reported outcomes must receive equal prominence: Clavien–Dindo grading capturing 30- and 90-day events; continence reported using both pad-free and social-continence definitions with baseline status; and sexual function, bowel symptoms, fatigue, pain, HRQoL, and treatment burden. IP5-MATTER demonstrates that patients may trade survival and progression differently against incontinence and fatigue, so a statistically significant oncologic endpoint may not represent the preferred treatment [65].

### 9.3. Biomarker-Guided Selection

The central challenge is distinguishing a truly limited metastatic state from underdetected widespread disease. Candidate markers include quantitative PSMA PET burden and distribution, CTCs, circulating tumor DNA, genomic alterations, pathologic treatment response, PSA kinetics, and immune signatures [19,59,60,61]. Tumor-intrinsic biology may ultimately matter more than lesion count: aneuploidy and its candidate driver genes are associated with progression and metastasis in prostate cancer [84]; integrated multi-omic profiling of the immune microenvironment has yielded prognostic signatures that stratify survival and predict sensitivity to androgen-receptor-directed agents [85]; and the mechanisms of resistance to ADT and ARPIs—androgen-receptor amplification, mutation and splice variants, lineage plasticity, and neuroendocrine differentiation—define the biology that any local treatment must contend with [86]. The aggressive-variant signature identified in the trial by Chapin and colleagues illustrates how such markers could enter selection [36]. None of these markers has been validated to predict benefit from prostatectomy specifically, and prognostic association must not be mistaken for a treatment interaction.

Translational sampling around RARP is a unique opportunity, but should be embedded in randomized trials, or biomarker changes after surgery may be misread as clinical benefit.

### 9.4. Molecular Imaging and Image-Guided Surgery

PSMA PET/CT will increasingly determine eligibility, but trials should report both conventional and molecular stage, since guidelines and historical randomized evidence rest largely on conventional imaging. Standardized reporting and central review would reduce heterogeneity.

PSMA-targeted radioguided or fluorescence-guided surgery may improve intraoperative localization of nodal disease; consensus work supports prospective evaluation but does not establish improved survival or justify routine use in OMPC [87,88]. It may improve the completeness of selected nodal resections but cannot eradicate microscopic systemic disease and should be tested as a surgical-quality intervention within a broader oncologic trial rather than promoted as a substitute for systemic treatment.

### 9.5. Cost-Effectiveness and Health-Technology Assessment

No formal economic evaluation of cytoreductive prostatectomy in OMPC has been published, and the question cannot be answered until an effect on survival or quality-adjusted survival is established. The relevant inputs are nonetheless identifiable. Robotic surgery carries higher capital and consumable costs than open or laparoscopic prostatectomy, partly offset by shorter hospitalization, and its cost-effectiveness in localized disease depends strongly on center volume [89]. In the metastatic setting, potential offsets include avoided local interventions, catheterization, and palliative pelvic RT, whereas additional costs include perioperative complications, incontinence management, and the likelihood that systemic and metastasis-directed treatment continue regardless of surgery. Prostate RT, the guideline comparator, is comparatively inexpensive and increasingly hypofractionated. Health-technology-assessment bodies increasingly require such analyses before adopting surgical innovations, and trials funded through such programmes, of which PRESIDENT is an example, are well placed to embed prospective health-economic evaluation. Future surgical trials should collect resource-use data alongside clinical and patient-reported outcomes.

## 10. Conclusions

The role of cytoreductive prostatectomy in OMPC has moved from a technically exploratory concept to an active randomized research question. Registries, institutional series, and prospective cohorts establish that the operation—in contemporary practice most often performed as RARP—can be delivered to carefully selected patients, may reduce local complications, and provides complete pathologic information.

RAMPP provides the first randomized prostatectomy-specific CSM signal (5-year CSM 13% versus 23%; HR 0.39, 95% CI 0.16–0.98) but does not close the question: early termination, a small number of events, no significant OS benefit, similar clinical progression, a control arm that predates modern systemic intensification, and 14% major surgical morbidity all demand caution, and a second randomized trial with a predominantly surgical local-therapy arm found no progression or survival advantage. No trial has tested, or demonstrates, a robot-specific oncologic effect. Meta-analyses remain dominated by low-certainty, overlapping observational evidence.

Accordingly, cytoreductive prostatectomy, including cytoreductive RARP, should remain investigational. Current EAU guidance recommends modern combination systemic therapy, prostate RT for eligible low-volume de novo disease, and no surgery for M1 disease outside clinical trials [7]. The appropriate present-day use of RARP is therefore in expert centers, preferably within prospective trials, after modern staging, multidisciplinary review, and explicit counseling about uncertainty, functional morbidity, the RT alternative, and the likely need for continued systemic and metastasis-directed treatment (Figure 1).

The next generation of studies must ask if prostatectomy delivers meaningful net benefits beyond contemporary systemic therapy and RT, identify patients with true differential benefit, standardize surgical quality, and measure outcomes that matter to patients.

## 11. Key Points

- OMPC is heterogeneous; lesion count alone is insufficient for surgical selection.
- Systemic therapy remains the treatment backbone, and prostate RT is guideline-supported for selected low-volume de novo metastatic disease.
- RAMPP supplies the first randomized prostatectomy-specific CSM signal (HR 0.39, 95% CI 0.16–0.98) but did not demonstrate a statistically significant OS benefit and reported 14% major surgery-related morbidity; a separate randomized phase II trial with a predominantly surgical local-therapy arm showed no progression or survival benefit. Neither trial tested a robot-specific effect.
- The Dai phase II trial supports radical treatment of the primary tumor but combined RP and RT and cannot isolate a surgical effect.
- PSMA PET/CT improves staging and may refine selection, but proposed quantitative thresholds remain unvalidated.
- The most plausible current benefits of cytoreductive prostatectomy are local control and complete pathology; continence, sexual function, and treatment burden must be weighed against them.
- Current EAU guidance restricts surgery for M1 disease to clinical trials; cytoreductive prostatectomy, whatever the approach, should be concentrated in expert centers and prospective protocols.

Abbreviations: ADT, androgen deprivation therapy; ARPI, androgen receptor pathway inhibitor; CI, confidence interval; CSM, cancer-specific mortality; CTCs, circulating tumor cells; EAU, European Association of Urology; ECOG, Eastern Cooperative Oncology Group; ePLND, extended pelvic lymph-node dissection; HR, hazard ratio; HRQoL, health-related quality of life; MDT, metastasis-directed therapy; mHSPC, metastatic hormone-sensitive prostate cancer; OMPC, oligometastatic prostate cancer; OS, overall survival; PCa, prostate cancer; PFS, progression-free survival; PSA, prostate-specific antigen; PSMA, prostate-specific membrane antigen; PSMA PET/CT, prostate-specific membrane antigen positron emission tomography/computed tomography; PSMA-TV, PSMA-derived tumor volume; RARP, robot-assisted radical prostatectomy; RP, radical prostatectomy; RT, radiotherapy; SABR, stereotactic ablative body radiotherapy; SBRT, stereotactic body radiotherapy; SEER, Surveillance, Epidemiology, and End Results.

## Figures and Tables

**Table 1:** Key evidence informing cytoreductive prostatectomy and RARP.

Key Limitations	Principal Contribution	Intervention/Comparator	Design/Population	Study
Small, nonrandomized, highly selected, pre-PSMA era.	Early direct evidence that carefully selected patients could undergo prostatectomy.	Cytoreductive RP after favorable response vs. systemic management.	Feasibility and case–control; low-volume skeletal metastases.	Heidenreich et al. [26]
Selection bias, older systemic era, modest sample.	RARP-specific comparative oncologic signal.	RARP plus systemic therapy vs. ADT alone.	Retrospective; 79 men with bone oligometastases.	Jang et al. [27]
Nonrandomized and selected; important negative/balancing evidence.	No clear survival benefit; fewer locoregional complications.	Cytoreductive RP vs. no surgery.	Prospective case–control; low-volume bone metastases.	Steuber et al. [28]
Patient/clinician treatment choice; residual confounding.	Feasibility, functional and local-event data in a prospective program.	Surgery, prostate RT, or no local treatment.	Prospective multicenter/registry cohorts.	LoMP studies [30,31]
Not powered for efficacy.	Established feasibility of recruitment, randomization, and specialist-center surgery.	Systemic therapy plus RP vs. systemic therapy.	Randomized feasibility trial; 51 men.	TRoMbone [34]
Mixed local modalities; cannot isolate surgery.	Improved radiographic PFS; supports primary-tumor treatment.	ADT plus radical local therapy vs. ADT; local therapy in 96/100 (85 RP, 11 RT); crossover to local therapy in 17/100 controls.	Phase II randomized trial; 200 men.	Dai et al. [35]
Single arm, expert centers, short follow-up, treatment heterogeneity.	Contemporary perioperative, continence, and intermediate-term outcome estimates.	Cytoreductive RP within multimodal treatment.	Multicenter PSMA PET-staged cohort; 116 men.	Rajwa et al. [32]
Early closure (132 of 452 planned); 21 CSM events; no significant OS benefit (81% vs. 74%); similar clinical progression (59% vs. 60%); grade ≥III complications in 9/66 (14%); control arm largely ADT alone; not RARP-specific.	Lower 5-year CSM (13% vs. 23%; HR 0.39, 95% CI 0.16–0.98); first prostatectomy-specific randomized efficacy signal.	Best systemic therapy plus RP vs. systemic therapy alone.	Randomized efficacy trial; 132 men with 1–5 bone metastases.	RAMPP [37]
Underpowered; surgery and RT combined; 26/180 (14%) progressed before randomization; ARPI in only 9/119; not RARP-specific.	Only randomized trial with a predominantly surgical local-therapy arm; no PFS (HR 0.89, 95% CI 0.59–1.34) or OS benefit.	Systemic therapy plus local treatment (surgery 45/60, RT 13/60, none 2/60) vs. systemic therapy alone.	Randomized phase II; 119 of 180 enrolled men (66%) randomized after 6 months of systemic therapy.	Chapin et al. [36]
Pilot endpoints only; in the radical arm 5/26 underwent prostatectomy, 14/26 received RT and 7/26 received no intervention.	Randomization feasible; complete pathological response in 6/53 (11%), indicating that a viable primary tumor persists after systemic therapy.	Standard care vs. minimally invasive ablative therapy vs. radical treatment, each with stereotactic ablative body radiotherapy (SABR) to metastases.	Randomized phase II pilot; 81 of 108 eligible men (75%) randomized.	IP2-ATLANTA [41]

**Table 2:** Practical, explicitly investigational selection framework for cytoreductive prostatectomy (delivered as RARP in most centers).

Features Generally Arguing Against Surgery	Features Requiring Caution	Features Supporting Consideration	Domain
Visceral metastases; extensive bone disease; rapid new lesions.	Borderline low-volume disease; numerous PSMA-only lesions; uncertain burden classification.	1–5 bone or nonregional nodal lesions; no visceral disease; stable or responding burden.	Disease burden
Rectal fixation, disproportionate morbidity, or no credible local-control benefit.	Bulky T3/T4 disease; bladder-neck or ureteric involvement requiring complex reconstruction.	Resectable; plausible negative margins or meaningful symptom prevention.	Primary tumor
Progression or treatment resistance despite appropriate systemic therapy.	Short observation interval or discordant PSA and imaging response.	Deep and sustained PSA/imaging response to guideline-concordant systemic therapy.	Systemic response
Poor performance status, short life expectancy, inability to resume systemic treatment.	Frailty, major comorbidity, poor baseline continence, competing mortality.	ECOG 0–1; long life expectancy; acceptable operative risk; informed preference.	Patient factors
Diffuse or biologically aggressive pattern inconsistent with an oligometastatic state.	PSMA-only stage migration; high PSMA-TV; nonregional nodal burden [60,61].	Limited, treatable PSMA PET burden; no occult widespread disease.	Imaging/biology
Routine surgery outside specialist oversight or without systemic-treatment planning [7].	Uncertain sequencing or unavailable MDT/RT expertise.	High-volume robotic center, multidisciplinary plan, prospective protocol or registry.	Treatment environment

## Data Availability

No new data were created or analyzed in this study. Data sharing is not applicable to this article.

## Supplementary Materials

The following supporting information can be downloaded at https://www.mdpi.com/article/10.3390/cancers18193207/s1, Table S1: Complete literature and trial-registry search strategy; Table S2: Oligometastatic states, their classification basis, and implications for cytoreductive prostatectomy.
