# Supplementary material for: Cytoreductive Radical Prostatectomy in Oligometastatic Prostate Cancer and the Role of the Robotic Platform: A Narrative Review
Source: Cancers (Basel). 2026 Oct 4;18(19):3207. doi: 10.3390/cancers18193207 (PMC13664479; doi:10.3390/cancers18193207)
Supplement: Supplementary file 1 [file cancers-18-03207-s001.zip › cancers-4586685-supplementary.pdf]

Supplementary Material

Cytoreductive Radical Prostatectomy in Oligometastatic Prostate Cancer and the Role of the Robotic Platform: A Narrative Review (cancers-4586685). Reference numbers correspond to the reference list of the revised main manuscript.

Supplementary Table S1. Complete literature and trial-registry search strategy

Source	Search string / approach	Limits	Date
PubMed/MEDLINE	("oligometastatic" OR "oligo-metastatic" OR "low-volume" OR "low volume" OR "low-burden" OR "limited metastatic" OR "metastatic hormone-sensitive" OR "metastatic castration-sensitive" OR "Neoplasm Metastasis") AND ("Prostatic Neoplasms" OR prostate cancer OR prostatic) AND ("Prostatectomy" OR "robot-assisted" OR robotic OR "Robotic Surgical Procedures" OR cytoreductive OR cytoreduction OR "primary tumor" OR "primary tumour" OR "local therapy" OR "local treatment")	English; inception to search date	1 July 2026
PubMed/MEDLINE (context searches)	As above AND ("PSMA" OR "positron emission tomography"); AND ("metastasis-directed" OR SBRT OR "stereotactic"); AND ("quality of life" OR continence OR "patient-reported"); AND (abiraterone OR enzalutamide OR apalutamide OR darolutamide OR docetaxel OR intensification)	English; inception to search date	1 July 2026
Guidelines	EAU Guidelines on Prostate Cancer (2026 edition), sections on M1 hormone-sensitive disease and local treatment	—	23 June 2026
ClinicalTrials.gov	Condition: metastatic prostate cancer; Intervention/other terms: prostatectomy OR cytoreductive OR "local therapy" OR "definitive treatment"; all statuses	Interventional studies	1 July 2026
ISRCTN registry	Free text: prostatectomy metastatic; cytoreductive prostatectomy	—	1 July 2026
EU Clinical Trials Register / CTIS	Free text: prostatectomy AND metastatic prostate cancer	—	1 July 2026

Records were screened by title/abstract and full text by two authors (P.G.S., J.G.R.). Because this is a narrative review, no formal risk-of-bias scoring or quantitative synthesis was performed and no protocol was registered; evidence was prioritized as described in Section 2 of the main text.

Supplementary Table S2. Oligometastatic states, their classification basis, and implications for cytoreductive prostatectomy

Clinical state	Classification basis	Operational description	Relevance to cytoreductive prostatectomy	Key interpretive issue	Refs
De novo synchronous OMPC	Timing of metastasis relative to diagnosis (ESTRO/EORTC state)	Limited metastatic disease detected at initial PCa diagnosis.	Principal population studied for cytoreductive RP; untreated primary tumor remains present.	Best evidence applies mainly to fit patients with bone-predominant, non-visceral disease.	26,34,35,37
Metachronous oligorecurrence	Timing of metastasis relative to prior local treatment (ESTRO/EORTC state)	Limited metastases after previous definitive local treatment.	RP is relevant only when the prostate remains in situ; after prior RT this becomes salvage surgery.	Cannot be pooled with de novo surgical cohorts because biology, prior treatment, and morbidity differ.	3,4
Oligoprogression	Behaviour during systemic therapy (ESTRO/EORTC state)	Progression at a limited number of sites during otherwise controlled systemic therapy.	Primary RP is rarely the dominant question; MDT or a change of systemic therapy usually has greater immediate relevance.	Local treatment of the prostate is unlikely to control resistant systemic clones.	3,68
Low-volume mHSPC (CHAARTED)	Disease-volume definition from a systemic-therapy trial (absence of visceral metastases and of ≥4 bone metastases with ≥1 outside the vertebral bodies and pelvis)	Burden category defined by trial criteria rather than a strict lesion count.	Provides the strongest randomized context for primary-tumor RT and current guideline recommendations.	Low volume is not synonymous with oligometastatic, and RT evidence cannot be assumed to prove a surgical effect.	7–9
Low-burden mHSPC (STAMPEDE)	Disease-volume definition from a prostate-RT trial (CHAARTED-type criteria applied to the STAMPEDE population)	Same criteria as CHAARTED low volume; the term is retained in this review only when STAMPEDE is quoted.	Population in which prostate RT improved OS; benchmark population for any surgical strategy.	Term differs but definition overlaps CHAARTED; both were established on conventional imaging.	8,21
Lesion-count OMPC (1–3 or 1–5 lesions)	Numerical threshold used in surgical and MDT studies	Limited number of metastatic lesions, threshold varying by study.	Eligibility criterion for RAMPP (1–5 bone metastases), TRoMbone and most cRP cohorts.	Thresholds are not interchangeable between studies; distribution and site matter as much as number.	26,34,37
PSMA PET-defined OMPC	Imaging modality used to define the state	Limited disease detected using molecular imaging, often with occult lesions missed conventionally.	May improve selection and operative planning, including ePLND; basis of PRESIDENT eligibility.	Stage migration complicates comparison with trials staged by CT and bone scan.	4,14,32

cRP, cytoreductive radical prostatectomy; ePLND, extended pelvic lymph-node dissection; MDT, metastasis-directed therapy; mHSPC, metastatic hormone-sensitive prostate cancer; OMPC, oligometastatic prostate cancer; OS, overall survival; PCa, prostate cancer; PSMA PET, prostate-specific membrane antigen positron emission tomography; RP, radical prostatectomy; RT, radiotherapy. The "classification basis" column distinguishes states defined by disease taxonomy (timing/behaviour of metastases), by disease-volume criteria from trials, by lesion count, and by imaging modality; the same patient may belong to more than one category.
